# Implications of Altered Endosome and Lysosome Biology in Space Environments

**DOI:** 10.3390/ijms21218205

**Published:** 2020-11-02

**Authors:** Ian R. D. Johnson, Catherine T. Nguyen, Petra Wise, Daniela Grimm

**Affiliations:** 1 Research in Space Environments Group, UniSA Clinical and Health Sciences, University of South Australia, Adelaide, SA 5000, Australia; ngucy037@mymail.unisa.edu.au; 2 Department of Hematology and Oncology, Children’s Hospital of Los Angeles, Los Angeles, CA 90027, USA; pwise@chla.usc.edu; 3 Department of Microgravity and Translational Regenerative Medicine, Clinic for Plastic, Aesthetic and Hand Surgery, Otto-von-Guericke-University Magdeburg, 39106 Magdeburg, Germany; dgg@biomed.au.dk; 4 Department of Biomedicine, Aarhus University, 8000 Aarhus C, Denmark

**Keywords:** lysosomes, spaceflight, autophagy, bone, muscle, differentiation

## Abstract

Space exploration poses multiple challenges for mankind, not only on a technical level but also to the entire physiology of the space traveller. The human system must adapt to several environmental stressors, microgravity being one of them. Lysosomes are ubiquitous to every cell and essential for their homeostasis, playing significant roles in the regulation of autophagy, immunity, and adaptation of the organism to changes in their environment, to name a few. Dysfunction of the lysosomal system leads to age-related diseases, for example bone loss, reduced immune response or cancer. As these conditions have been shown to be accelerated following exposure to microgravity, this review elucidates the lysosomal response to real and simulated microgravity. Microgravity activates the endo-lysosomal system, with resulting impacts on bone loss, muscle atrophy and stem cell differentiation. The investigation of lysosomal adaptation to microgravity can be beneficial in the search for new biomarkers or therapeutic approaches to several disease pathologies on earth as well as the potential to mitigate pathophysiology during spaceflight.

## 1. Introduction

Endosomes and lysosomes are critical to the function of every cell in the human body. They are dynamic organelles that mediate adaptation of cell metabolism to environmental cues [1,2]. With a low pH that regulates hydrolase function, lysosomes are fundamental to the degradation and recycling of cellular material and participate in a multitude of cellular processes such as signalling, cell adhesion, gene regulation, immunity, migration and plasma membrane repair [1]. Endosomes and lysosomes are also essential for autophagy, removal and recycling of cellular compartments and are critical to immune function [3,4,5]. They are also critical for intercellular signalling; the biogenesis of late endosomes to multivesicular bodies provides a source of extracellular vesicles, known as exosomes, that are enriched for cell-specific proteins and mRNA that can affect target cells through altered protein translation or other signalling events [6]. Disruption to the endo-lysosomal system can significantly affect cell homeostasis and impact tissue and organ function; for example, lysosomal dysfunction contributes to age-related disorders such as Parkinson’s and Alzheimer’s diseases, neurodegenerative disorders (e.g., mucopolysaccharidosis) and cancer [7,8,9].

Onset of disease pathologies, namely those associated with ageing, can be accelerated in extreme environments such as in space [10,11,12]. Human physiology is significantly altered in this environment resulting from stressors such as hypergravity (>1 g), microgravity (<1 g) and ionising radiation [13]. These induce physiological changes and disease phenotypes similar to those that are observed in ageing-related processes, such as bone atrophy, sarcopenia, accumulative oxidative stress and diminished immune function [14,15,16,17]. Indeed, many of the physiological alterations that astronauts exhibit in low-gravity conditions may be attributed to autophagy or alterations in the endo-lysosomal system [18,19,20,21].

First described from the physiological changes observed in rat cardiomyocytes on Bion 4 [22] and salivary glands of rats on-orbit in Spacelab 3 [23], there is a growing body of work associating altered autophagic responses to spaceflight or microgravity [21]. Many of the autophagic and lysosomal alterations are summarised in Table 1. These results are derived from observations of alterations to in vitro cell culture systems or the direct action of tail suspension (hindlimb unloading) in rodents on muscle/bone. Interestingly, non-exhaustive meta-analysis of mRNA microarray datasets from space-flown animals or cell lines show alterations in expression of endo-lysosomal or vesicular trafficking genes. For example, significant differential expression of endosome associated proteins such as Rab5 (early), Rab7 (late) and Rab11 (recycling) are observed in space flown mice (GSE94381 [24]) and mesenchymal stem cells (GSE100930 [25]), whilst various kinesins, dyneins or dynactins required for lysosomal trafficking also show significant differential expression. Further evidence of altered endo-lysosomal function during spaceflight is suggested by the near two-fold expression change of transcription factor EB (TFEB; GSE94381), a critical regulator in lysosomal biogenesis. Understanding the role of endosomes, lysosomes and autophagic processes in cellular function and disease pathogenesis in space environments may reveal novel physiological biomarkers and new therapeutic targets or countermeasures against space-related stressors and ageing-related pathologies.

## 2. The Endosome-Lysosome System and Autophagy in Microgravity-Induced Pathophysiology

### 2.1. Bone Loss

**Figure 1:**
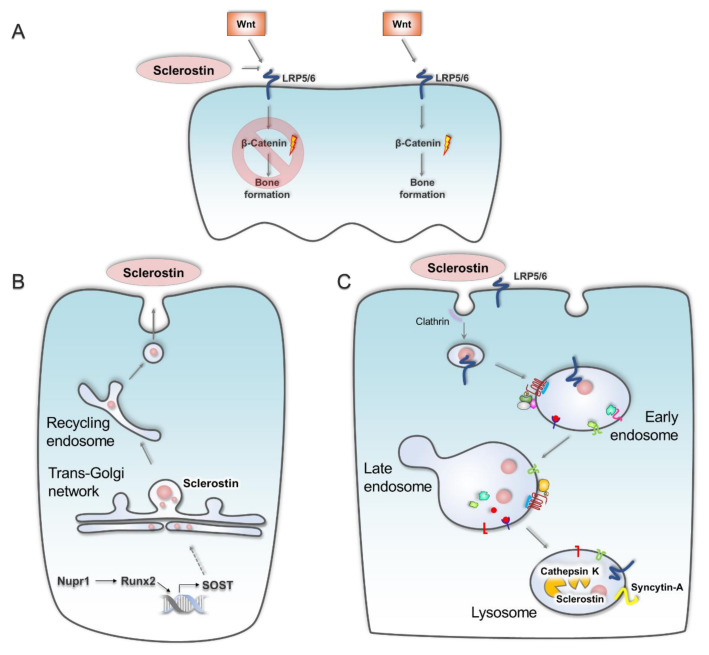
Sclerostin inhibits Wnt signalling through competitive binding to LRP5/6 (**A**) and requires endo-lysosomal pathways for its secretion (**B**) and degradation (**C**).

The absence of load bearing forces affects living organisms at the macro and microscopic levels, resulting in significant physiological changes that often present as pathologies within bone and muscle [12,52,53]. Astronauts lose approximately 10–20 % of their bone mass in microgravity [54,55], mimicking bone atrophy in joints and vertebrae of patients with severe osteoporosis [56,57,58]. Bone loss rates are skeletal site-specific and range approximately 1% in thoracic vertebrae and approximately 6% in spine to over 8% at the femoral neck or proximal femur after only six months in orbit [59]. Skeletal homeostasis requires a balanced action of osteoclasts for bone resorption and osteoblasts for bone formation. Both are critical in bone mass renewal, remodelling and bone growth [60,61,62,63]. Bone loss results from altered osteocyte function within the bone matrix; osteocytes deeply embedded within the mineralised matrix of bone sense mechanical stimuli (mechano-sensation) [56,57,58], and alter their surrounding environment via mechano-transduction to maintain skeletal homeostasis [64]. Skeletal unloading, such as during bed rest, upregulates the expression of sclerostin [65,66], an essential Wnt signalling inhibitor (Figure 1A), and reduces bone formation [67]. In microgravity-simulated conditions of reduced hindlimb loading in rats and osteocyte culture in rotating wall vessels, osteocytes express and secrete increased amounts of sclerostin [62,63,68], inhibiting bone remodelling and regeneration through reduced osteoblast proliferation and differentiation [21]. Like many other secretory proteins, sclerostin secretion and uptake may be tightly integrated with the endosome pathway, with its secretion mediated by vesicular trafficking from the biosynthetic pathway (Figure 1B) and its uptake by clathrin-dependent endocytosis into the endo-lysosomal network (reviewed by Suen and Qin [69]) and degraded by lysosomal hydrolase cathepsin K (Figure 1C) [70]. Whilst secretion is elevated by reduced mechanical load, disruption to endo-lysosomal biogenesis and altered hydrolase function may inhibit the efficient endocytosis and degradation of sclerostin, maintaining its inhibitory effects on Wnt signalling pathways. Interestingly, sclerostin expression is induced by Nupr1 via Runx2 transcription factor [71]. Nupr1, a stress-associated gene, is highly expressed in mature osteoclasts; however, Nupr1-deficient mice show elevated autophagy and apoptosis [71], suggesting that Nupr1 is a protective factor in osteoclast homeostasis or that osteoclasts pre-disposed to elevated Nupr1 will survive spaceflight stressors that induce autophagy and apoptosis. Investigations on the interplay of stress response, autophagy, and the endo-lysosomal system on sclerostin and osteoclast activity in space environments may reveal new insights to maintaining bone health in spaceflight.

The endosome-lysosome system has significant roles in osteoblast and osteoclast homeostasis through recycling and degradation of material, cell differentiation and signalling [72,73]. The organelles have critical roles in regulating signal transduction of membrane-bound receptors such as the parathyroid hormone receptor, which affects osteoclasts as well as proximal tubular cells of kidney [73,74,75]. Stimulation by parathyroid hormone in spaceflight increases the release of Ca^2+^ from bone, inducing nutrient deprivation of osteoclasts [76,77] and increased autophagic activity and lysosome-mediated degradation of the bone under microgravity conditions [73,78].

**Figure 2:**
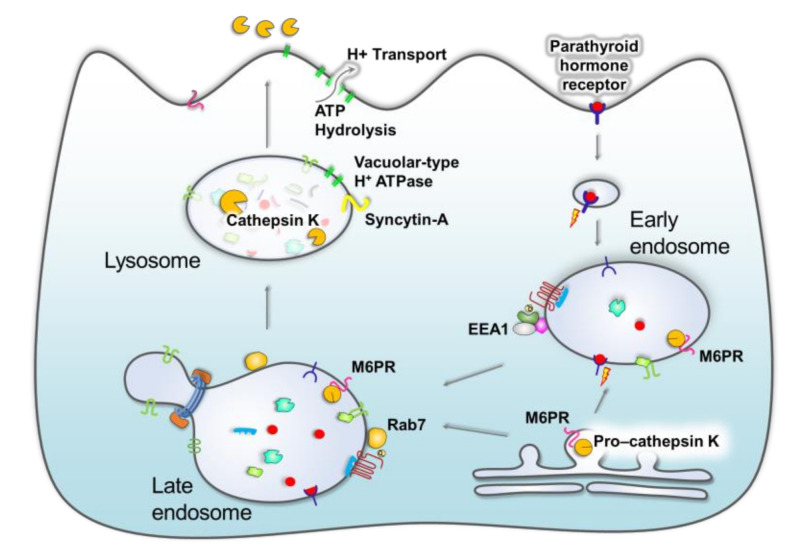
The endo-lysosomal system is essential to bone maintenance through secretion of lysosomal hydrolases and efficient uptake and signalling from endosomal compartments of ligands such as parathyroid hormone. Maturation of lysosomes and plasma-membrane fusion releases lysosomal content to the bone surface and enriches the plasma membrane with proton pumps that enhance acidification. Rab7 is a critical protein in biogenesis of early endosome to late endosomes, and overexpression may increase targeting of lysosomes to the ruffled border. Mannose-6-phosphate receptor (M6PR) may affect cathepsin K trafficking and secretion, inducing aberrant hydrolysis of extracellular proteins. Arrows depict potential biogenesis and trafficking pathways of secretory and endocytosed proteins.

Lysosomes are essential to the maintenance of bone through acidification of the bone surface, where lysosomal hydrolases are released by exocytosis that demineralises bone matrix [39,79,80]. In microgravity conditions, there is both an increase in osteoclast resorption through pit formation [52] and a decrease in osteoblast synthesis and bone regeneration [59,81,82,83]. Secretion of lysosomal content acidifies the bone surface, degrading the lacuna and enabling resorption of the bone [84]. The ruffled borders of osteoclasts are enriched with vacuolar-type H^+^ ATPase through binding of lysosomes to the membrane, enhancing acidification through active proton transport and ATP hydrolysis across the plasma membrane (Figure 2). Differential expression of endosome proteins such as the late endosome marker Rab7 observed in spaceflight [24,25] significantly impairs the resorption capacity [4], since these proteins are integral to cell polarisation, ruffled border and osteoclast bone resorptive capability [85,86]. Furthermore, expression of mannose-6-phosphate receptor (M6PR) is elevated during spaceflight [87] which disrupts lysosome-mediated osteoclast activity through increased M6PR-mediated cathepsin K lysosomal hydrolase secretion and osteoclast resorption [41,87]. This may have deleterious effects on the activity of sclerostin and its inhibitory effects on the Wnt signalling pathways discussed above.

Restoring the balance of lysosomal exocytosis and cell polarisation may improve bone regeneration under microgravity conditions and overall bone health [73]. Ethiraj et al. [5] showed that microgravity induces the syncytin-A expression in RAW264.7 preosteoclast cells without RANKL stimulation. The syncytin-A expression co-localised with lysosomes in preosteoclast cells and acridine orange staining showed RANKL elevated autophagy activity (see Figure 2). Moreover, siRNA suppression of syncytin-A clearly reduced the autophagy activity in the cells. The authors suggested that targeting the syncytin-A expression as an interesting countermeasure to reduce bone loss in space [5]. In a recent study Ethiraj et al. [88] also demonstrated that proteasome inhibition via MG-132 treatment abolishes RANK expression in preosteoclast cells. The authors reported that MG-132 also supresses microgravity-induced elevated osteoclast differentiation and bone resorption, further supporting the hypothesis of RANK involvement in the process. Similarly, protease inhibition has been shown to suppress K48-linked poly-ubiquitin proteins, suggesting that the ubiquitylation of proteins plays a role in suppression of RANK receptor expression in preosteoclast cells [88].

Furthermore, elevated osteoclast formation coincides with increased expression of Atg5 and LC3-II autophagic markers, autophagosome formation and the upregulation of inflammatory cytokines in microgravity environments [4]. As shown in Table 1, there are significant number of studies in simulated and real microgravity that display autophagic markers such as LC3 and Atg proteins and increased autophagosomes depicted by increased puncta. Autophagy is integrally linked to the endo-lysosomal system, utilising many of the same machinery. Recently, Nakamura et al. described how activation of LC3 by lipid attachment is essential for autophagosome formation [89]. Interestingly, lipidated LC3 activates TFEB, a critical transcription factor for lysosome biogenesis and autophagy [90]. The mechanism of this upregulation remains to be elucidated; however, the elevated expression of TFEB in microgravity [24] and evidence of LC3-mediated TFEB activation strongly suggests that lysosomal biogenesis is altered and that autophagy and stress response has a role in these processes.

Expression of S100A8, a calcium binding protein that has pivotal roles in autophagy, is significantly upregulated in microgravity and may contribute to the increased cathepsin K activity observed in microgravity-exposed mouse bone marrow-derived preosteoclast cells [41]. This suggests that modulation of autophagy and lysosomal function may remediate the increased osteoclast bone resorptive ability, osteoclast differentiation and reduced bone formation observed in spaceflight. The biogenesis of the endo-lysosomal network is tightly controlled by expression of Rab GTPases such as Rab7 and transcription factors such as TFEB. Indeed, exosomes formed through maturation of late endosomes may be critical to bone homoeostasis (Reviewed by Gao et al. [91]); thus, endos-lysosomal biogenesis may have significant consequences in osteoclastogenesis and bone resorption, highlighting the need to investigate their roles of bone remodelling during spaceflight. Additionally, autophagy and exosome biogenesis seem to be directly linked via shared organelles and molecular machinery. A report from Guo et al. [92] presents a mechanism where autophagy-related proteins like ATG5 and ATG16L1, among others, are directly involved in the regulation of the fate of multivesicular bodies and latterly exosome biogenesis [92]. Autophagic machinery has been observed within exosomes and suggest a strong link between autophagy and exosome biogenesis (reviewed by Gudbergsson [93]). Indeed, recent initial analyses hint to substantial changes in exosome release and distribution of subtypes in cell culture supernatants collected from a controlled experiment on the International Space Station (ISS) in comparison to the ground control (unpublished data, from personal conversation). This leads to the question about the modifications in cell–cell communication via a possible adaptation of the exosomal payload or simply an altered dose response to the micro-vesicular transfer of information and the possible involvement of the wider endo-lysosomal system in this adaptive reaction.

### 2.2. Muscle Atrophy

Mitochondria are essential to the correct function of myofibre and muscle cells, and regulation of metabolism in these cells are significantly affected by autophagic processes [3]. Remodelling of the mitochondrial network, selective removal of mitochondria via autophagy (mitophagy) or mitochondrial reactive oxygen species (ROS)-activated autophagy may lead to the accumulation of dysfunctional and damaged mitochondria that contributes to myofibre and muscle degradation [94,95]. These processes can be induced by factors including cancer, ageing [96], and disuse [97], and are frequently observed in immobile elderly populations and in patients undergoing prolonged bed rest recovery [58,98]. Muscle atrophy resulting from altered protein turnover rates can be detected by elevated urinary amino acid excretion [77,99]. During the first 28 days on-orbit in Skylab missions SLS1 and SLS2, the average urinary amino acid concentration from atrophying muscles increased by 55 % and proportionate to the time spent in orbit [100]. Proteolysis requires lysosomal hydrolases such as Cathepsin L, that are shown to be upregulated in the muscle of rodents exposed to microgravity or hind-limb unloading experiments (Table 1). Without countermeasures, microgravity-induced mitophagy increases the number of slow type I muscle fibres and reduces fibre diameter and myofibril size, eliciting significant muscle atrophy [58,101,102]. Alterations to muscle structure and function primarily affects leg extensor muscles and postural muscles though a reduction in fibre size [58], pennation angle and sarcomere length [12,18], which may be attributed to lysosome-mediated mitophagy. These processes require expression of specific machinery such as Bnip3 and Bnip3L that bind LC3 and recruit autophagosomes to mitochondria [103]; indeed, inhibiting Bnip3 can reduce muscle wastage [104]. Interestingly, Bnip3 is downregulated in microgravity [105], suggesting an impairment of mitophagy with significant deleterious effects to muscle homeostasis, and a potential accumulation of damaged and dysfunctional mitochondria [95]. Moreover, Ma et al. suggest that in TFEB-overexpressing Bnip3-positive cells, more autolysosomes, and an increased ratio of lysosomes compared to autophagosomes are observed that may restore autophagosome processing and attenuate Bnip3-induced cell death [106]. TFEB is emerging as a critical factor in mitochondrial quality control and maintaining metabolic homeostasis; indeed, skeletal muscle TFEB-deficient mice have been shown to have impaired muscle energy balance and mitochondrial homeostasis-related genes are downregulated [107]. The critical role of TFEB in cell homeostasis (provided in a comprehensive review by Wang et al. [108]) suggests that it may play a pivotal role to cell response of microgravity and may be a targetable candidate for therapeutic countermeasures.

Stressors such as inflammation or starvation invoke muscle proteolysis through Forkhead Box (Fox) O transcription factor that is pivotal to the activation of autophagy-lysosome pathways [109]. FOXO3 overexpression has been shown to increase protein degradation via lysosomes in atrophied myoblast cells [110]. Whilst there is evidence of FOXO activation of lysosomal degradation and autophagy, the targets of these mechanisms must be elucidated. More detail on molecular mechanisms of muscle atrophy, including FOXO action, are described in a recent review by Ehmsen and Hoke [111]. Interestingly, in microgravity-exposed C. elegans, DAF-16, a FOXO ortholog, displays both elevated expression, and increased nuclear localisation in microgravity, suggesting that lysosomal activity is increased [112]. Lysosomal activity plays a significant role in the longevity of *C. elegans* [113], with evidence found recently that lysosomes may be critical to mitochondria signalling via lipid pools that enhance mitochondrial function and organism health [114]. Unilateral lower limb suspension does not appear to affect the expression of these in male humans [115], suggesting that microgravity plays a wider role in muscle atrophy via changes in lysosomal activity. In rodents exposed to real or simulated microgravity (see Table 1 for references), soleus muscle and myocardia display significant autophagy activity, ranging from increased numbers of autophagosomes and autophagy-related proteins, upregulated expression and activity of lysosomal hydrolases to ubiquitination to altered distribution of lysosomes (detected by lysosomal tripeptidyl peptidase) to the sarcoplasmic reticulum. The close association of lysosomes with the sarcoplasmic reticulum suggests a role of calcium signalling from lysosomes that may enhance calcium release from the sarcoplasmic reticulum that may initiate autophagic responses [116,117]. The role of calcium in autophagy is beyond the scope of this review; however, Bootman et al. review the intrinsic link between calcium and the pro- and anti-autophagic effects that exist in cells [118]. The role of lysosomes in mitochondrial function/regulation and their importance in age-related disorders that are enhanced during spaceflight highlight the importance of investigations of lysosomal function and autophagy during spaceflight that may provide new insight or countermeasures against muscle dysfunction in ageing and immobile populations.

### 2.3. Cell Differentiations

Cell differentiation is regulated by autophagy and lysosomal signalling [119] and many studies have uncovered associations of endosome-lysosome proteins with stem cell function. For example, lysosome-associated Rab GTPase Rab44 negatively regulates osteoclast differentiation when overexpressed [120]. Indeed, its depletion affects lysosomal pH and lysosomal Ca^2+^ influx that is critical for osteoclast differentiation [120,121]. The functions of Rab44 have yet to be fully elucidated and may be of significance to changes observed in spaceflight. Villegas et al. have recently reported that mouse embryonic stem cell differentiation is driven by a change in distribution of the lysosomal transcription factor TFE3 from the nucleus, where it promotes stem cell renewal, to the cytoplasm where lysosomal Rag GTPases inactivates TFE3 and subsequently promotes cell differentiation [122]. mTORC1 protein complex is critical to this process; in its role in cell response to environmental cues, activation of mTORC1 deactivates TFE3. Inactive mTORC1 results in TFE3 translocation to the nucleus that induces autophagy and upregulation of lysosome target genes, and thus, the reduced phosphorylation or downregulation of mTOR observed in simulated microgravity studies (Table 1), for example Myoblast C2C12 cells or HUVEC endothelial cells. A recent review details the requirement of autophagy in stem cell differentiation [123]. Indeed, this suggests that, in spaceflight, cell differentiation will be significantly altered—significant changes to the expression of autophagic machinery occur in microgravity that may critically affect stem cell populations and affect broad aspects of human physiology [85,124], for example, the ability to regenerate and repair damaged tissues and to maintain the haemopoietic system. In microgravity, stem cell precursors accumulate in bone marrow though a partial inhibition of the progenitor-to-differentiated cell transition through cell cycle arrest and activation of cellular quiescence and senescence [85,125]. Osteoblast differentiation may also be affected through morphological alterations autophagy and lysosomal degradation [52,53,126]. Microgravity may therefore create a broad inhibition of stem cell-based tissue regeneration, significantly affecting long-term survival in space [19].

Microgravity may provide new avenues to study stem cell differentiation and to investigate disease pathogenesis. Through cumulative mitochondrial damage, increased autophagy and stem cell clearance, and downregulation of cell signalling pathways, loss of cell stemness is induced in this environment [119,125]. The cytoprotective mechanisms of autophagy against stresses such as oxidative stress and DNA damage prevent chromosomal instability, and these may both inhibit and promote tumourigenesis [123,127]. In cancer stem cells starved of oxygen and nutrients, autophagy is upregulated and promotes cancer survival, decreasing the effectiveness of anticancer treatments [123]. Interestingly, under simulated microgravity conditions, cancer cell lines differentiate into two phenotypes [125]; one growing adherently on the bottom of the culture flask and one that assembles into spheroids or organoids. The phenotypes may develop from autophagic pathways initiated in stem-like cells that are present in previously assumed “homogenous” cell lines [128], and provide in vitro conditions to study the effects of cancer treatments on patients, providing significant benefits to the study of heterogenic diseases such as cancer [125,126]. Thus, investigation of spheroid or organoid development in microgravity may elucidate the role of autophagy in disease pathogenesis.

### 2.4. Oxidative Metabolism

Spaceflight promotes significant oxidative stress, and reactive oxygen species (ROS) production may reduce the metabolic rate of muscle and increase the rate of bone loss in microgravity [129], decreasing longevity of astronauts in spaceflight [130]. Oxidative stress is a consequence of microgravity-induced changes to cells and those of cosmic radiation in the form of protons and heavy ions (e.g., Fe, Si and O). These particles result in damage to proteins, lipids and DNA [131,132], and may induce oxidative stress either through activation of autophagy to remove damaged cell components or directly through linear transfer of energy from these particles and stripping of electrons from atoms and molecules producing free radicals within the cell [133].

There is increasing evidence to suggest that ROS activates genes-promoting tumour metastasis [134]. In the Mir and LMS missions, Stein et al. observed elevated 8-OH-dG levels during spaceflight compared to bedrest controls [135] that may be a result of microgravity or increased ionising radiation that significantly affects mitochondrial electron transport chains, affecting ROS production [136]. ROS production enhances muscle and bone atrophy during spaceflight, creating changes to the cytoskeletal structure, altering adhesion molecule expression, integrins and actin-associated proteins that could result in vascular ageing [137]. Using simulated microgravity, Ran et al. found increased ROS in mouse embryonic stem cells [138]. Yun et al. have recently published a comprehensive review of the role of autophagy in oxidative stress, detailing the changes occurring in mitochondria [139]. Mitochondria are a major source of cellular ROS and are frequently damaged or become dysfunctional in microgravity [95]. In turn, this may induce accumulation of enlarged endo-lysosomal structures and impairment of lysosomal acidification and activity [140]. Enlargement of endo-lysosomal structures are regulated by TRPML1, an ion channel that resides in late endosome and lysosome compartments [140]. Activation of TRPML1 from elevated ROS induces the release of lysosomal Ca^2+^ to the cytosol and disruption to osteoclastogenesis [121,141]. TRPML1-dependent Ca^2+^ release also induces a signalling cascade mediated by TFEB, increased autophagy, and elimination of damaged mitochondria and excess accumulation of ROS [141].

Whilst ROSs are small enough to diffuse through the cell, their localisation to specific compartments activate receptors and initiate specific redox signalling responses [142]. Endosomal compartments are vital sources of NADPH oxidase (NOX)-generated ROS species that are vital to initiating autophagic responses to oxidative stress [143,144]; endosome-localised NOX2 enzymes catalyse NADPH reduction that induces mitochondrial and nuclear DNA damage and apoptosis [138,145]. ROS-generating endosomes (also known as redoxosomes) can augment signalling of endocytosed receptors that can induce mitochondrial dysfunction-induced apoptosis and from lipids such as C2-ceramide [146,147]. NOX-generated ROSs have critical roles in oxidative inhibition of protein tyrosine phosphatases (PTPs) that are vital to insulin response. Interestingly, insulin resistance is observed during spaceflight [148,149] that may be attributed to an imbalance of mitochondrial and NOX-generated ROS. Mitochondrial and cytoplasmic ROS are finely balanced in response to glucose and insulin. H_2_O_2_ generated by endosomal NOX oxidise and inactive PTPs such as PTEN supresses the development of insulin resistance (reviewed in [150]). Endosome and lysosome function may determine subcellular response to ROS during spaceflight and may provide pathways to target aberrant ROS production, reducing detrimental outcomes of spaceflight including targeting insulin resistance in long duration spaceflight.

### 2.5. The Lysosomal System as a Monitor for Physiological Stress

Spaceflight induces significant pathophysiological changes to the body resulting from stresses at the cellular level. In addition to the involvements of the endo-lysosomal system detailed above, in response to microgravity, human physiology may be critically affected by changes to microbiomes and altered immune responses. Understanding the effects of microgravity on immunosuppression is important in spaceflight, as potential biomarkers may be released from the endo-lysosomal system and may provide a means for targeting through development of new pharmacological interventions for diseases [151]. For example, the pro-inflammatory cytokine IL-1β is located within lysosomes and is a potent activator of osteoclastogenesis which enhances resorption. Downregulation of IL-1β indicates impairment of the osteoclastogenesis activation pathway and inhibition on bone formation seen in spaceflight [152]. Suppression of IL-1β signalling occurs through autophagic activation of p62 degradation via the lysosomal pathway, causing ubiquitinated protein aggregates and proinflammatory responses [153]. IL-1β may be used as a potential biomarker for spaceflight-induced stress, and investigation of its role in response to spaceflight may further advance our understanding of various diseases such as neurodegeneration and muscle disorders [153].

Multiple cathepsins such as cathepsin L have been shown to be related to accelerated muscle atrophy in microgravity; in vitro, upregulation of cathepsin L degrades myofibrillar proteins leading to muscle atrophy [154]. Cathepsin L was shown to be upregulated in microgravity [41], making it a potential biomarker for muscle atrophy analysis [154] or lysosomal function in general.

Endosomal NOX2 may further serve as a potential biomarker for stress and countermeasure response, as its elevation in spaceflight contributes to oxidative stress [155,156]. The accumulation of NOX2 correlates with increased ROS production, increasing endothelial cell proliferation [145]. There is a dearth of knowledge regarding NOX2 that must be addressed; inhibition of this enzyme also has immunosuppressive potential [155], and altered endo-lysosomal function may significantly affect NOX2 activity, changing the critical ROS balance required for correct cell function. The study of endosomes and lysosomes in space environments may reveal many more pathways to provide countermeasures to long-term spaceflight and to an ageing population on Earth.

## 3. Discussion and Conclusion

Lysosomes are acidic single-membrane-bound cytoplasmic organelles containing soluble hydrolytic proteins enabling the degradation of material from the endocytic and autophagic pathways. They remove unnecessary or dysfunctional cell components and proteins to maintain a balance of synthesis, degradation and recycling for proper cell differentiation and organ development. Disruption to lysosomal function can have significant deleterious effects to human development and may be critical to cancer pathogenesis. As such, the changes to lysosomal function induced by both microgravity and ionising radiation may be a significant factor in increasing cancer risk from spaceflight. Indeed, altered differentiation of cells in microgravity affects macrophage maturation, further impacting cancer pathogenesis. 

**Figure 3:**
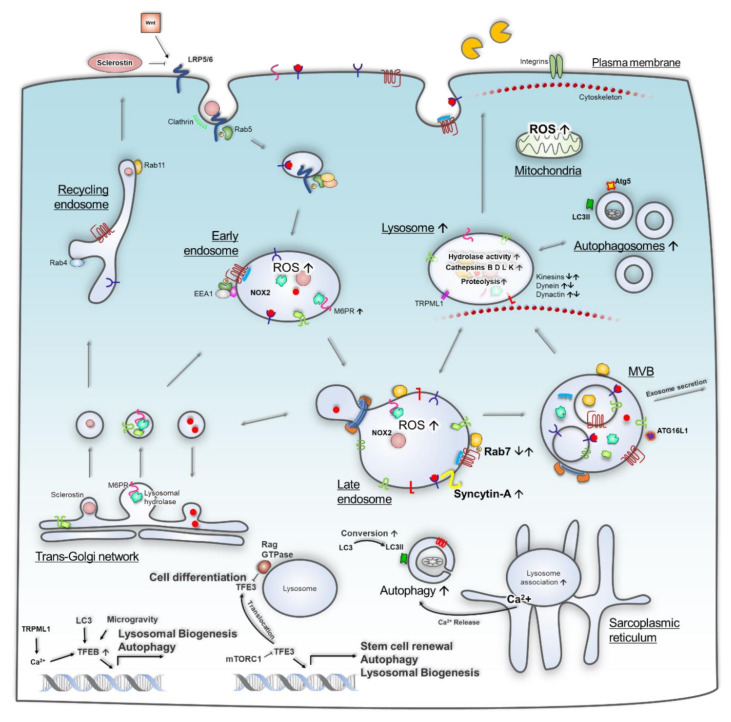
The endo-lysosomal system is a complex pathway that is vital to countless processes in the cell, and its dysfunction results in the hallmarks of many diseases. Shown here are some of the findings from studies detailed in this review, revealing many alterations to cell biology in microgravity that are associated with endosomes and lysosomes. Further research into alterations of the endo-lysosomal system in space may provide substantial new knowledge in its involvement in disease pathogenesis to provide new biomarkers or therapeutic targets. ↓↑ depicts up- and downregulation observed in spaceflight or associated analogues. Grey arrows depict potential lysosomal biogenesis trafficking pathways. Black arrows depict action/downstream effects on proteins and pathways.

The complexity of the lysosomal system is depicted in Figure 3, showing a fraction of the involvement of endosomal/lysosomal functions in cells that are affected in space environments, whether through their involvement in secretion of Wnt inhibitors or hydrolases or their roles in ROS production or calcium signalling resulting in increased autophagy. Stressors experienced in spaceflight such as microgravity change cell homeostasis with effects frequently resembling ageing such as loss of muscle and bone mass. The study of many systems has relied on cell lines, which may already have a propensity for a particular biological pathway that may not reflect true biological responses. Likewise, the exposure to radiation in cyclotrons may not reflect real radiation either on-orbit or during spaceflight to Mars. Clinostat or Random positioning machine (RPM) microgravity simulation may also produce shear forces and physical response of cells not observed during spaceflight. However, it is encouraging that many autophagic responses are observed in both real and simulated microgravity both in cell cultures and animals.

Each biological system affected by these stressors is closely reliant on endosome or lysosome function, whether through secreted hydrolases that degrade bone matrix or through autophagic response and recycling of macromolecules. Autophagy and lysosomal activity have been shown to be elevated in both simulated and real space environments, with increased numbers of autophagosomes observed as early as the 1980s. These may be a response to temporary collapse of the cytoskeleton and initiation of starvation responses resulting from failed endocytosis and trafficking resulting in increased autophagic gene expression and activity. Long-term studies on the lysosomal system may reveal new candidate biomarkers for monitoring physiological stress and responses to therapeutic countermeasures, such as when reducing intracellular ROS. These countermeasures may need to target the endo-lysosomal system to regulate autophagic responses, to minimise muscle and bone loss or to minimise deleterious effects on stem cells that are critical to, amongst others, gut function. To examine these systems, secreted biomarkers such as within extracellular vesicles (e.g., exosomes) or soluble lysosomal proteases can be collected. Sufficient quantities can be obtained through long-term culture in simulated microgravity (e.g., RPMs). Antibody-based detection of specific markers with microfluidic devices may enhance this analysis. Technology development here may be implemented to on-orbit experiments and be readily translatable to detection of biomarkers in blood plasma. Significant endo-lysosomal biology can be realised using techniques such as confocal laser scanning microscopy. Samples may be readily fixed in real or simulated microgravity or radiation fields and analysed later. Live cell imaging adds an important dimension to the investigation of lysosome biology; however, implementation of this must achieve enough resolution—lysosome vesicles can be as small as 50 nm in diameter. Furthermore, analysis of lysosome and endosome trafficking may require long-term imaging in microgravity environments that are only suitable for on-orbit systems. To reach these goals of providing real-time cell biology analysis, in June 2020, Yokogawa’s CSU-W1 Confocal Scanner unit arrived in the Japanese Experiment Module of the ISS. Likewise, the FLUMIAS (Fluorescence-Microscopic Analyses System for Life-Cell-Imaging in Space) spinning disk confocal laser fluorescence microscope represents a new imaging capability for live cell imaging experiments on suborbital ballistic rocket missions [157,158]. Thiel et al. reported the first successful operation of the FLUMIAS-DEA, a miniaturized high-resolution 3D fluorescence microscope on the International Space Station (ISS) [159]. The available real-time analysis technology on the ISS will extend the current knowledge about the dynamics of cellular reactions and adaptations to microgravity in the future.

In conclusion, through investigations of the endosome and lysosome system and its role in response to physiological stresses in space environments, we may obtain greater understanding of its importance in human development and disease pathogenesis, with potential to provide better diagnostic biomarkers to disease onset and potential targets for therapeutic countermeasures.

## Figures and Tables

**Table 1:** The effect of spaceflight or simulated environments on autophagy and the endo-lysosomal system.

Physiological, Protein, Gene or Organelle Change	Effect	Organism/Model System	Radiation or Simulated/Real µg	Ref.
**Lysosomes, phagosomes**	Increased	Purkinje cells, Rat	Radiation (291 Mev/nucleon, 100–300/mm^2^)	[26]
**Autophagosomes**	Increased	Myocardia, Rat	Microgravity (Real)	[22]
**Lysosomal activity**	Elevated	Bone	Microgravity (Real)	[27]
**Autophagic vacuoles, endocytic vesicles, lysosomes**	Increased number, size	Salivary gland, Rat & Mouse	Microgravity (Real)	[23,28]
**Lysosomal tripeptidyl peptidase**	Altered distribution	Skeletal muscle, Rat	Microgravity (Real)	[29]
**Sarcoplasmic reticulum (SR)**	Localise to myofibril disruptions – may release Ca^2+^, may induce autophagy			
**Glutathione**	Decreased	Liver, mouse	Microgravity (Real)	[19]
**Lipid membrane metabolism, endocytosis, inflammatory pathways**	Enriched			
**MuRF-1, MAFbx**	Increased expression	Soleus and EDL muscle, mice	Microgravity (Real)	[30]
**LC3β, CTSL**	Unchanged			
**IGF-1 and IL-6**	Increased in EDL, Decreased in Soleus			
**c-Fos**	Upregulated	Supraoptic neurons, Rat	Microgravity (Real)	[31]
**Vasopressin**	Downregulated			
**Autophagosomes, lysosome-like bodies**	Increased	Osteocytes and Soleus myotendinous junction, Rhesus monkey	Microgravity (Real)	[32,33]
**Lactate dehydrogenase type A**	Mono-ubiquitinated	Myoblastic L6 cells, Rat	Microgravity (Simulated, tail suspension)	[34]
**Ubiquitinated protein & MAFbx, Murf-1, Nedd4, XIAP**	Upregulated expression	Soleus Muscle, Rat	Microgravity (Simulated, hindlimb suspension)	[35,36,37]
**Glucose metabolism**	Increased response upon muscle atrophy			
**Proteolysis**	Increased (Ca^2+^ regulated from SR?)			
**Lysosomal and Ca^2+^-dependent proteolysis**	Enhanced activity			
**Cathepsin B, D, L**	Upregulated expression			
**N-acetylglucosaminidase (lysosomal)**	Increased activity	Gastrocnemius & soleus muscle, CD1 mice	Microgravity (Simulated, hindlimb suspension)	[38,39]
**Bcl-2, Bax**	Increased expression ratio			
**Vps34, Beclin-1, Cathepsin D**	Upregulated	Cardiac tissue, rat	Microgravity (Simulated, tail suspension)	[40]
**p62**	Downregulated			
**mTOR**	Decreased phosphorylation (s2448)			
**Ca^2+^**	Oscillates	HEK293, Myoblast C2C12, Mouse bone marrow, RAW 264.7 osteoclast progenitor, MC3T3-E1, HepG2, colorectal, HUVEC, L-540, HDLM-2 cell lines	Microgravity (Simulated, clinostat & RWV)	[4,5,41,42,43,44,45,46,47]
**LC3 +ve autophagosomes**	Increased puncta (72 h)			
**LC3-II**	Increased conversion from LC3-I			
**AMPK, T172, ULK1, ATF4, Beclin-1**	Increased phosphorylation			
**AMPK S485**	Reduced phosphorylation at 24 h only			
**ATG12, ATG5, ATP1A1, ATP5A1, Bcl-2, MnSOD, Cu/ZnSOD, Mcl-1, mTOR, Rab7, GPCR, P58**	Downregulated			
**ATG14, ATG16L1, ATG4B, ATG7, ATG5, Bax, Beclin-1, Ire1α, LC3, MnSOD, p62, Syncytin-A Tnfsf10, ULK1, MITF, s100a8, CREB, CXCL4**	Upregulated			
**P62**	Reduced at 72h			
**ROS**	Elevated			
**Cytoskeleton**	Temporary collapse of microvilli at 24 h & altered microtubule/actin remodelling	TCam-2 seminoma cell line	Microgravity (Simulated, RPM)	[48,49]
**LC3 +ve autophagosomes**	Increased			
**LC3-II**	Increased conversion from LC3-I			
**Ca^2+^, ROS**	Elevated temporarily at 24 h			
**MnSOD, Casp9, BECN1**	Downregulated	NB-1 neuroblastoma cell line	X-ray, Microgravity (Simulated, clinostat)	[50]
**VDAC2**	Upregulated			
**Vps15, Atg14L, Beclin1, p62, FOXO3A**	Downregulated	Endometrial stromal cells	Microgravity (Simulated, RPM)	[51]
**LC3BII**	Decreased			

## Abbreviations

µg	Microgravity
ROS	Reactive oxygen species
TFEB	Transcription factor EB
RPM	Random positioning machine
RWV	Rotating wall vessel
SR	Sarcoplasmic reticulum
EEA1	Early Endosome Antigen 1
M6PR	Mannose-6-phosphate receptor
TRPML1	Transient receptor potential cation channel 1
